# Neurogenic Pulmonary Edema Associated with Underlying Lung Disease after a Breakthrough Seizure

**DOI:** 10.1155/2012/560942

**Published:** 2012-08-02

**Authors:** Gaurav Dutta, Spiro Demetis

**Affiliations:** Department of Internal Medicine, Lutheran Medical Center, 150 55th Street, Brooklyn, NY 11209, USA

## Abstract

Neurogenic pulmonary edema (NPE) can result from various central nervous system disorders such as brain malignancies, traumatic brain injuries, infections, and seizures. Although the pathogenesis is not completely understood, NPE creates an increase in pulmonary interstitial and alveolar fluid. It has been reported with prolonged seizure activity. Treatment for NPE is largely supportive. If unrecognized, it can lead to hypoxia and respiratory arrest. We report a case of NPE in a middle-aged female patient following a breakthrough seizure in whom an immunological cause for respiratory findings was high on the differential list, based on her past medical history and chronicity of symptoms. Rapid symptomatic and radiological improvement following hospitalization led to the correct diagnosis.

## 1. Case Report 

**Figure 1:**
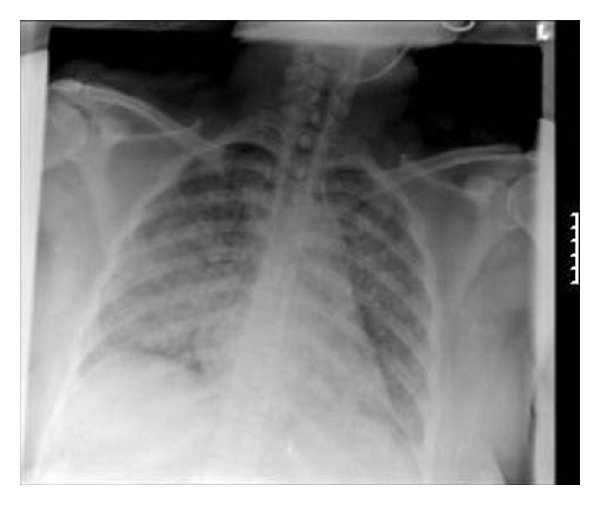
Chest radiograph on admission showing bilateral interstitial disease with alveolar filling (worse on the right side).

**Figure 2:**
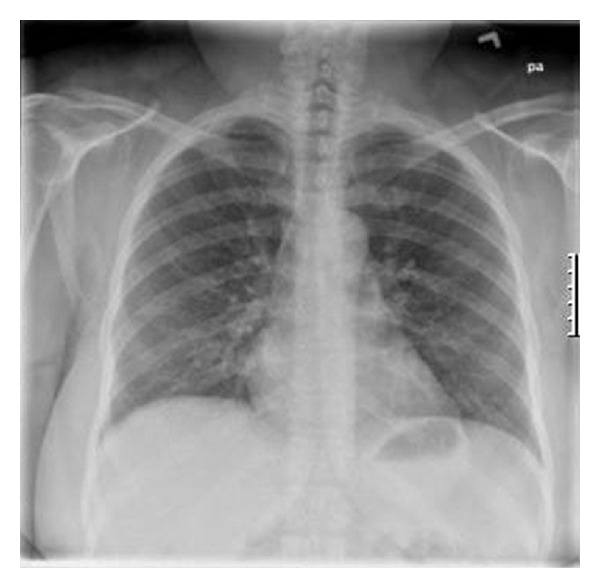
Chest radiograph on day no. 3, a near normal study with marked radiological resolution of interstitial disease.

A 50-year-old female presented to the ER of an inner city teaching hospital after experiencing a generalized seizure episode. She had a medical history significant for immune thrombocytopenia for 30 years and seizure disorder since experiencing a right occipitoparietal stroke at the age of 47 years, for which she took lacosamide and aspirin. She reported being allergic to sulpha drugs. She had smoked 1/2 packs per day for 30 years but quit 3 years before. She reported feeling out of breath after walking less than a block and a persistent cough without much expectoration for the past 8 to 10 months. Upon hospitalization, she received supplemental oxygen in addition to her home medications. A chest radiogram done on admission (Figure 1) showed bilateral patchy infiltrates; pulse oximetry done the day after showed oxygen saturation of around 94% on room air dropping to 90% after a 6-minute walk. A repeat chest radiogram done on the third hospital day (Figure 2) revealed significant clearance of the infiltrates with much improvement in her oxygen saturation values both at rest and with ambulation. Cardiac markers and 2D echocardiogram were normal. The patient did not experience any more seizures during hospitalization and was discharged on the fourth day to be followed up in the office. The diagnosis of NPE was made based on rapid resolution of the symptoms.

## 2. Discussion

Neurogenic pulmonary edema (NPE) can result from various central nervous system disorders such as brain malignancies, traumatic brain injuries, infections, and seizures. Epileptic seizures are the most common cause of NPE [1, 2]. Several case series reported that up to one-third of patients with fatal status epilepticus had clinical evidence of NPE [2, 3], while an autopsy study found that 87 percent of patients with epilepsy and unexplained sudden death had NPE [4]. It is uncertain whether NPE was the proximate cause of death in these studies [5]. NPE due to epileptic seizures generally occurs during the postictal period, and it may occur repeatedly in a given individual [1, 6, 7]. Although NPE was first reported following seizure activity in 1908 [8], NPE following seizure remains an underrecognized diagnosis with a poorly understood pathogenesis. All cases are characterized by an increase in extravascular lung water following a neurological insult. There is rapid flooding of the alveoli with protein-rich fluid. The mechanism for NPE likely involves increased pulmonary capillary permeability combined with massive centrally mediated sympathetic discharge resulting in elevated pulmonary vascular resistance [9–12].

The so-called NPE trigger zones include the hypothalamus and the medulla, specifically area A1, A5, nuclei of solitary tract, and the area postrema [13]. Area A1 is located in the ventrolateral aspect of the medulla and is composed of catecholamine neurons, which project into the hypothalamus [13]. The neurons from area A5, located in the upper portion of the medulla, project into the preganglionic centers for spinal cord sympathetic outflow [13]. These areas are related to respiratory regulation and receive input from the carotid sinus. Unilateral stimulation of the area postrema also results in profound hemodynamic changes, including increased cardiac output, peripheral vascular resistance, and hypertension [13]. The presence of hypothalamic lesions among NPE patients confers a worse prognosis [14].

Most cases of NPE present with nonspecific signs and symptoms such as dyspnea, tachypnea, tachycardia, and respiratory insufficiency. Signs and symptoms develop rapidly following the neurological insult, usually within minutes to hours [15]. Patients may also have frothy pink sputum or rales. Chest radiography will frequently demonstrate bilateral diffuse alveolar infiltrates, although unilateral NPE has been reported in the adult literature. Most NPEs resolve rapidly. More than one-third of adult patients had resolution of symptoms within 24 hours, with greater than 75% resolving within 7 days [15]. In the appropriate clinical situation, diagnosing NPE may obviate the need for further diagnostic testing and evaluation of other etiologies of respiratory insufficiency. Also, if NPE is more common in already diseased lung is a question for further research, the point being that a diseased lung having an already smaller reserve would decompensate with smaller insults. In other words, having a healthy lung would render protection against NPE. 

Despite more than a millennium of scientific experiments and case descriptions, the diagnosis and management of NPE remains controversial, and the entity remains underdiagnosed and underappreciated. The exact pathophysiology of NPE is still debated, and the wide variety of clinical situations in which it occurs can obscure diagnosis. The sudden development of hypoxemic respiratory failure following a catastrophic CNS event, which cannot be attributed to other causes of ARDS, is the only universally agreed upon characteristic of NPE. A common denominator in all cases of NPE is likely a surge in endogenous serum catecholamines that may result in changes in cardiopulmonary hemodynamics and Starling forces. It appears that the specific clinical manifestations of this surge may vary depending on the individual circumstance. In some patients, cardiac dysfunction may predominate; in others, capillary leak is the primary manifestation. These patterns have obvious implications for the diagnosis and treatment of individual cases.

In keeping with the previous discussion, our patient had a preexisting lung condition and thus likely decompensated below her baseline because of a worsened A-a gradient caused by capillary leak. She improved once the latter resolved, representing a slightly atypical presentation of NPE.
